# A Model for the Coexistence of Competing Mechanisms for $\text {Ca}^{\text {2}+}$ Oscillations in T-lymphocytes

**DOI:** 10.1007/s11538-024-01317-w

**Published:** 2024-06-13

**Authors:** Paco Castaneda Ruan, J. Cory Benson, Mohamed Trebak, Vivien Kirk, James Sneyd

**Affiliations:** 1 Department of Mathematics, University of Auckland, Auckland Central, 1142 Auckland New Zealand; 2 Department of Pharmacology and Chemical Biology, University of Pittsburgh School of Medicine, Pittsburgh, Pennsylvania 1526 USA; 3 Vascular Medicine Institute, University of Pittsburgh School of Medicine, Pittsburgh, Pennsylvania 1526 USA; 4 Graduate Program in Cellular and Molecular Physiology, The Pennsylvania State University College of Medicine, Hershey, Pennsylvania 17033 USA

**Keywords:** Calcium, Lymphocytes, Mathematical model, Bifurcation analysis

## Abstract

$\text {Ca}^{\text {2}+}$ is a ubiquitous signaling mechanism across different cell types. In T-cells, it is associated with cytokine production and immune function. Benson et al. have shown the coexistence of competing $\text {Ca}^{\text {2}+}$ oscillations during antigen stimulation of T-cell receptors, depending on the presence of extracellular $\text {Ca}^{\text {2}+}$ influx through the $\text {Ca}^{\text {2}+}$ release-activated $\text {Ca}^{\text {2}+}$ channel (Benson in J Biol Chem 29:105310, 2023). In this paper, we construct a mathematical model consisting of five ordinary differential equations and analyze the relationship between the competing oscillatory mechanisms.. We perform bifurcation analysis on two versions of our model, corresponding to the two oscillatory types, to find the defining characteristics of these two families.

## Introduction

The role of calcium ions ($\text {Ca}^{\text {2}+}$) as an intracellular second messenger in cells has been studied since the late 1800s (Ringer 1883; Carafoli 2002). Regulation of the $\text {Ca}^{\text {2}+}$ concentration in the cytosol is known to control a wide array of cellular functions across a diverse range of cell types. As this regulation is central to cellular function, cells are equipped with a complex set of pumps, channels and exchangers designed to maintain a tight control of the cytosolic $\text {Ca}^{\text {2}+}$ concentration. $\text {Ca}^{\text {2}+}$ extrusion from the cytoplasm is done primarily through plasma membrane $\text {Ca}^{\text {2}+}$ ATPases (PMCA) and sarco/endoplasmic reticulum ATPases (SERCA) (Higgins et al. 2006; Michelangeli and East 2011; Wuytack et al. 2002). These pumps require ATP to transport $\text {Ca}^{\text {2}+}$ across their respective membranes and establish a steep concentration gradient. $\text {Ca}^{\text {2}+}$ entry into the cytoplasm occurs via $\text {Ca}^{\text {2}+}$ channels that allow the ions to flow down the electrochemical gradient. At the endoplasmic reticulum (ER) membrane, the two main $\text {Ca}^{\text {2}+}$ release channels are the inositol 1,4,5-trisphosphate receptor ($\text {IP}_{\text {3}}\text {R}$) and the ryanodine receptor (RyR) (Foskett et al. 2007; Kevin Foskett and Daniel Mak 2010); at the outer plasma membrane (PM), $\text {Ca}^{\text {2}+}$ channels are classified as receptor-operated (ROCC), store-operated (SOCC) or voltage-gated (VGCC), depending on their activation mechanism (Dupont et al. 2016; Thompson and Shuttleworth 2013; Catterall 2011; Yoast et al. 2020).

**Fig. 1:**
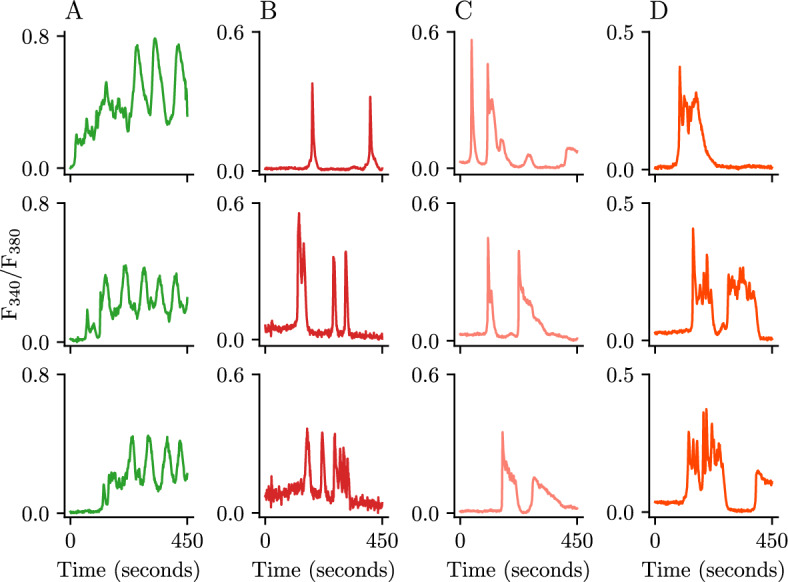
Experimental observations in Jurkat T-cells. Representative traces of Fura-2 imaging of $\text {Ca}^{\text {2}+}$ oscillations caused by TCR stimulation of Jurkat T-cells with 125 ng/mL $\alpha$-CD3. Cells in column A are stimulated in the presence of 2 mM extracellular $\text {Ca}^{\text {2}+}$; cells in columns B, C and D are stimulated in a $\text {Ca}^{\text {2}+}$-free environment

In T-lymphocytes, agonist stimulation by $\alpha$-CD3 induces two distinct types of $\text {Ca}^{\text {2}+}$ oscillations (Benson et al. 2023). When stimulated in a $\text {Ca}^{\text {2}+}$-rich environment, the cytoplasmic $\text {Ca}^{\text {2}+}$ concentration in these cells exhibits transient $\text {Ca}^{\text {2}+}$ spikes before transitioning to a wide, ‘sinusoidal-like’ oscillation (Fig. 1A). To sustain these oscillations, extracellular $\text {Ca}^{\text {2}+}$ is transported into the cytoplasm through the store-operated $\text {Ca}^{\text {2}+}$ Entry (SOCE) pathway via a SOCC located at the junction between the PM and ER membrane: the $\text {Ca}^{\text {2}+}$ release-activated $\text {Ca}^{\text {2}+}$ (CRAC) channel (Trebak and Kinet 2019; Dupont et al. 2016; Dolmetsch and Lewis 1994).

This $\text {Ca}^{\text {2}+}$ transport through CRAC has physiological relevance: in T-lymphocytes, translocation of the nuclear factor of activated T cells (NFAT) proteins from the cytoplasm to the nucleus, for example, is a pivotal step for cytokine production and correct immune function. $\text {Ca}^{\text {2}+}$ transported through this SOCC binds with the calmodulin protein to trigger the translocation of NFAT (Trebak and Kinet 2019; Srikanth and Gwack 2013). Hence, models of $\text {Ca}^{\text {2}+}$ oscillations in lymphocytes are usually concerned with CRAC activity above that of other channels (Dolmetsch and Lewis 1994; Naik 2020; Xf et al. 2008; Lewis 2011).

Replication of the same experiment in a $\text {Ca}^{\text {2}+}$-free environment, however, can lead to the appearance of a different class of oscillatory patterns including narrow $\text {Ca}^{\text {2}+}$ spikes (Fig. 1B), wide spikes (Fig. 1C) and wide spikes with intermittent bursting on a raised plateau (Fig. 1D) (Benson et al. 2023). The presence of $\text {Ca}^{\text {2}+}$ spikes in this scenario suggests that, in the absence of SOCE, a secondary oscillatory mechanism arises in this cell type, one related to fast $\text {Ca}^{\text {2}+}$ release from the ER, but that is typically ‘silenced’ by CRAC activity. Additionally, prior models of $\text {Ca}^{\text {2}+}$ oscillations have linked the appearance of $\text {Ca}^{\text {2}+}$ bursts and wide spikes to $\text {IP}_{\text {3}}$ production and decay and its corresponding effect on $\text {IP}_{\text {3}}\text {R}$ activity (Cloete et al. 2021).

These experimental results led  Benson et al. (2023) to suggest the existence of two fundamentally different kinds of oscillations during $\text {Ca}^{\text {2}+}$ signalling: $\text {IP}_{\text {3}}\text {R}$-mediated and CRAC-mediated. The $\text {IP}_{\text {3}}\text {R}$-mediated oscillations correspond to those observed in the $\text {Ca}^{\text {2}+}$-free environments, and the CRAC-mediated ones to the $\text {Ca}^{\text {2}+}$-rich environment. They supported this conclusion with a mathematical model capable of reproducing these two oscillatory types.

In this paper, we perform a more detailed study of the model presented in Benson et al. (2023) and show that it can reproduce the CRAC-mediated oscillations when extracellular $\text {Ca}^{\text {2}+}$ transport is considered, and the multiple types of $\text {IP}_{\text {3}}\text {R}$-mediated spikes when $\text {Ca}^{\text {2}+}$ influx is greatly decreased or removed. We show that the CRAC-mediated oscillations are sustained due to delayed $\text {Ca}^{\text {2}+}$ influx via CRAC, that the CRAC channel has a steep activation curve dependent on ER $\text {Ca}^{\text {2}+}$ concentration and that CRAC activity suppresses the $\text {IP}_{\text {3}}\text {R}$-based $\text {Ca}^{\text {2}+}$ spikes when present. After that, we show that the model is capable of reproducing all three types of $\text {IP}_{\text {3}}\text {R}$-mediated $\text {Ca}^{\text {2}+}$ spikes (narrow, wide, wide with an oscillatory plateau) depending on the level of $\text {IP}_{\text {3}}\text {R}$ stimulation as well as the level of the ER $\text {Ca}^{\text {2}+}$ stores.

**Fig. 2:**
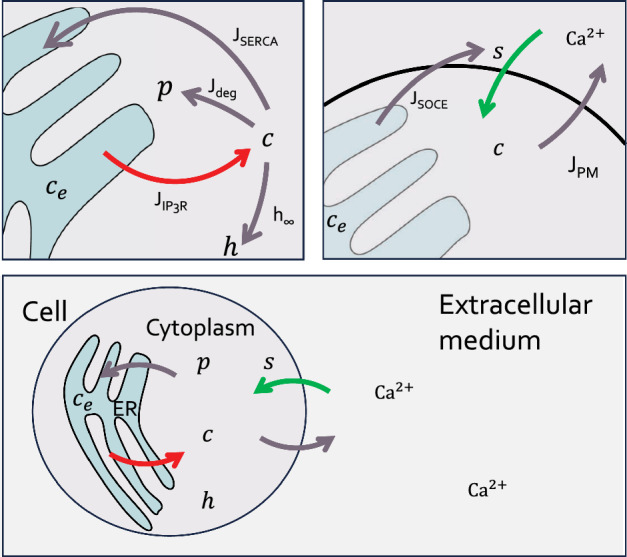
Schematic representation of the model. The bottom panel shows a schematic representation of the open-cell model (1). Top left panel shows a closeup of the model at the ER membrane. The red arrow represents the $J_{\text {IP}_{\text {3}}\text {R}}$ flux, transporting $\text {Ca}^{\text {2}+}$ from the ER into the cytoplasm; $\text {Ca}^{\text {2}+}$ is transported back into the ER via the $J_{\text {SERCA}}$ flux. Increases in cytoplasmic $\text {Ca}^{\text {2}+}$ lead to faster degradation of $\text {IP}_{\text {3}}$ ($J_{\deg }$), as well as a decrease in the rate of inactivation of the $\text {IP}_{\text {3}}\text {R}$, given by *h* ($h_\infty$). Top right panel shows a similar closeup of the model at the PM@. The green arrow represents $\text {Ca}^{\text {2}+}$ influx into the cytoplasm given by the variable *s*, and $\text {Ca}^{\text {2}+}$ removal from the cytoplasm is done via the $J_{\text {PM}}$ flux. A depleted ER (represented here with a faded color) sends a signal to the PM to initiate influx and refill the ER ($J_{\text {SOCE}}$) (Color figure online)

## Modeling

We model $\text {Ca}^{\text {2}+}$ transport in lymphocytes by using a set of five ordinary differential equations (ODEs). We include $\text {Ca}^{\text {2}+}$ influx through the CRAC channel. We model $\text {Ca}^{\text {2}+}$ release from the ER through the $\text {IP}_{\text {3}}\text {R}$ channel, as stimulation of the T-cell receptors (TCR) of this cell type is linked to increased $\text {IP}_{\text {3}}\text {R}$ activity (Trebak and Kinet 2019; Benson et al. 2023). Additionally, we assume that cytoplasmic $\text {Ca}^{\text {2}+}$ increases the rate of $\text {IP}_{\text {3}}$ degradation (Dupont et al. 2016). Finally, we include the effect of $\text {Ca}^{\text {2}+}$ extrusion from the cytoplasm via SERCA and PMCA@.

Let *c* and $c_e$, respectively, denote the $\text {Ca}^{\text {2}+}$ concentration in the cytoplasm and in the ER@; *h* is an auxiliary variable that controls the rate of $\text {Ca}^{\text {2}+}$-dependent inactivation of the $\text {IP}_{\text {3}}\text {R}$; *p* denotes the $\text {IP}_{\text {3}}$ concentration in the cytoplasm; *s* denotes the rate of $\text {Ca}^{\text {2}+}$ influx through the CRAC channel. The schematic version of the model is shown in Fig. 2. The model equations are

1a $$\begin{aligned} \frac{dc}{dt}&= J_{\text {IP}_{\text {3}}\text {R}} - J_{\text {SERCA}} + \delta (s - J_{\text {PM}}), \end{aligned}$$

1b $$\begin{aligned} \frac{dc_e}{dt}&= \gamma (J_{\text {SERCA}} - J_{\text {IP}_{\text {3}}\text {R}}) , \end{aligned}$$

1c $$\begin{aligned} \tau _h\frac{dh}{dt}&= h_\infty - h,\end{aligned}$$

1d $$\begin{aligned} \tau _p \frac{dp}{dt}&= V_\text {PLC}- J_\text {deg},\end{aligned}$$

1e $$\begin{aligned} \tau _s \frac{ds}{dt}&= J_\text {SOCE}- s. \end{aligned}$$

Here $J_{\text {IP}_{\text {3}}\text {R}}$ is the $\text {Ca}^{\text {2}+}$ flux out of the ER through the $\text {IP}_{\text {3}}\text {R}$s, $J_{\text {SERCA}}$ is the flux through a bidirectional SERCA pump, and $\delta J_{\text {PM}}$ is the flux through a unidirectional PMCA pump. The expressions for $J_{\text {IP}_{\text {3}}\text {R}}$, $J_{\text {SERCA}}$, $h_\infty$ and $J_{\text {PM}}$ are taken directly from Sneyd et al. (2017) and are reproduced in Appendix A, where default parameter values are also provided. A detailed construction of the $\text {IP}_{\text {3}}\text {R}$ model can be found in Dupont et al. (2016). The details of the $J_\text {deg}$ term are discussed in Sect. 2.1. The parameter $\gamma$ in (1b) is the volume ratio between the cytoplasm and the ER@.

To enable easy simulation of the $\text {Ca}^{\text {2}+}$-free scenario, we include an additional parameter $\delta$ in front of the *s* and $J_{\text {PM}}$ terms. $\delta$ is a measure of the rate of extracellular $\text {Ca}^{\text {2}+}$ exchange relative to the rate of the ER fluxes (Sneyd et al. 2004). The case when $\delta > 0$ is referred to as the *open-cell model*. When $\delta = 0$, $\text {Ca}^{\text {2}+}$ transport through the PM is not allowed and the model exhibits one conserved quantity: the total amount of free $\text {Ca}^{\text {2}+}$ inside the cell, given by $C_t = c + c_e/\gamma$ (Dupont et al. 2016; Sneyd et al. 2004). We exploit this observation by rewriting $c_e$ as a function of *c* and reducing the dimension of our system by one. By setting $\delta = 0$, *s* decouples from the rest of the system, further reducing the dimension of our system by one, giving us a set of three ODEs given by

2a $$\begin{aligned} \frac{dc}{dt}&= J_{\text {IP}_{\text {3}}\text {R}} - J_{\text {SERCA}} , \end{aligned}$$

2b $$\begin{aligned} \tau _h\frac{dh}{dt}&= h_\infty - h, \end{aligned}$$

2c $$\begin{aligned} \tau _p \frac{dp}{dt}&= V_\text {PLC}- J_\text {deg}. \end{aligned}$$

This version of the model is referred to as the *closed-cell model*. Since $c_e$ is not an explicit variable in this version the functional form of the $J_{\text {IP}_{\text {3}}\text {R}}$ and $J_{\text {SERCA}}$ fluxes is different to their open-cell counterpart; the closed-cell version of the fluxes and corresponding parameter values are given in Appendix A.

### $\text {IP}_{\text {3}}$ Dynamics

Binding of an agonist to a G protein-coupled receptor (GPCR) leads to the activation of a G protein, which in turn activates phospholipase C (PLC). PLC then acts on phosphatidylinositol bisphosphate (PIP_2_), cleaving it and producing both diacylglycerol (DAG) and $\text {IP}_{\text {3}}$. While PLC activity has been shown to depend on *c* in some cell types, this is not always the case, and for now we assume that the maximal rate of PLC activity ($V_\text {PLC}$) is determined solely by the level of agonist stimulation (Lee et al. 2023).

On the other hand, $\text {IP}_{\text {3}}$ can be metabolized into inositol 1,3,4,5-tetrakisphosphate (Ins (1,3,4,5) P_4_) by a $\text {Ca}^{\text {2}+}$-dependent 3-kinase (Dupont et al. 2016). The activity of this 3-kinase can be enhanced by the binding of the $\text {Ca}^{\text {2}+}$/calmodulin complex. Through this pathway, increases in cytoplasmic $\text {Ca}^{\text {2}+}$ concentration can then lead to faster metabolism (or degradation) of $\text {IP}_{\text {3}}$. This degradation is modelled by

3 $$\begin{aligned} J_\text {deg}&= V_\text {deg}\frac{c^2}{c^2 + K_\text {deg}^2} p, \end{aligned}$$

where $V_\text {deg}$ represents the maximal degradation rate. Finally, we consider that changes in $\text {IP}_{\text {3}}$ concentration occur on a timescale $\tau _p$.

### Slow CRAC Dynamics

Formation of the CRAC channel occurs upon ER depletion, when the $\text {Ca}^{\text {2}+}$ sensitive stromal interacting molecules 1 and 2 (STIM1, STIM2) in the ER membrane activate and bind with the $\text {Ca}^{\text {2}+}$ selective channels Orai1, Orai2 and Orai3 (Trebak and Kinet 2019; Srikanth and Gwack 2013; Dupont et al. 2016; Yoast et al. 2020; Emrich et al. 2021). The resulting STIM-Orai complex is the CRAC channel.

Experimental trials show that lymphocytes with genetic mutations that prevented the expression of both the STIM1 and STIM2 proteins, and thus were unable to activate the CRAC channel, did not show signs of ER refilling (or CRAC activity) under conditions of maximal ER depletion induced by blockage of SERCA pumps with thapsigargin (Tg), even in $\text {Ca}^{\text {2}+}$ rich environments. Cells that had been modified to exhibit only one variant of STIM1 or STIM2 did show a CRAC current and ER refilling, even if reduced when compared to the wildtype (WT) cells. Given this evidence, we consider that SOCE through the STIM-Orai channel is the only relevant pathway for extracellular $\text {Ca}^{\text {2}+}$ influx (Benson et al. 2023).

We model $\text {Ca}^{\text {2}+}$ transport through this ER-dependent channel using the phenomenological expression

4 $$\begin{aligned} J_\text {SOCE}&= \frac{V_\text {SOCE}}{1 + e^{s_1(c_e - K_e)}}, \end{aligned}$$

which is a sigmoidal function in $c_e$. Measurements of the CRAC current for T cells as a function of the ER $\text {Ca}^{\text {2}+}$ concentration result in a curve with a similar form (Luik et al. 2008). The parameter $s_1$ controls the steepness of the activation curve, while $K_e$ controls the midpoint. The maximal rate of transport through the channel is given by $V_\text {SOCE}$. We do not make a distinction between the contribution of STIM1 and STIM2 at this stage, and address this in Sect. 4.

Forced ER depletion via the use of Tg has been shown to induce oscillations (Dolmetsch and Lewis 1994; Benson et al. 2023). At the same time, experimental measurements have shown that increases in CRAC activity lag behind ER depletion, which suggests that the formation of the CRAC channel is slow in comparison to the ER depletion rate (Dolmetsch and Lewis 1994; Zweifach and Lewis 1995; Lewis 2011). This led Dolmetsch and Lewis to hypothesize that $\text {Ca}^{\text {2}+}$ influx via the CRAC channel and $\text {Ca}^{\text {2}+}$ release from the ER oscillate out of phase with each other (Dolmetsch and Lewis 1994). To model this phenomenon, we consider that *s* evolves on a timescale given by $\tau _s$. This timescale must be slow relative to the changes in the $\text {Ca}^{\text {2}+}$ variables, to generate the delayed CRAC response.

## Results

### The Model is Capable of Reproducing the Two Families of Oscillatory Patterns

**Fig. 3:**
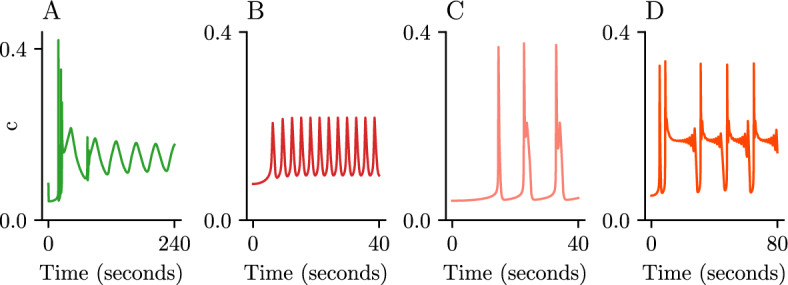
Types of $\text {Ca}^{\text {2}+}$ oscillations produced by the model. Cytoplasmic $\text {Ca}^{\text {2}+}$ traces obtained by simulation of the open-cell (1) (Panel **A**) and closed-cell models (2) (Panels **B**, C, **D**) using the values in Table 1. All simulations use initial values corresponding to the equilibrium solution for $V_\text {PLC}= 0$. Panel **A** uses parameter values $\delta = 2, V_\text {PLC}= 0.1$; Panel **B** uses $\delta = 0, V_\text {PLC}= 0.1, C_t = 140$; Panel C uses $\delta = 0, V_\text {PLC}= 0.1, C_t = 75$; Panel D uses $\delta = 0, V_\text {PLC}= 0.195, C_t = 95$. Panels **A** through **D** use different horizontal axes to increase visual clarity. Parameter values not specified here can be found in Table 1

We implement the open and closed-cell versions of (1) using XPPAUT (Ermentrout 2012) and the AUTO package (Doedel et al. 2021). Data processing and graph generation are done using the numpy 1.19.1 and matplotlib 3.4.0 Python packages (Harris et al. 2020; Hunter 2007). The code used to generate the figures can be found in github (0Huitzil 2023). Using the parameter values found in Table 1, model (1) is capable of producing the following types of oscillations:

1. CRAC-mediated: Parameter values $\delta = 2, V_\text {PLC}= 0.1$ $\mu$M/s. This choice simulates an open-cell environment and generates an attractor with an amplitude of $c \approx 0.2$ $\mu$M and oscillatory period of $T \approx 40$ seconds (Fig. 3A).
2. Narrow spike: Parameter values $\delta = 0, V_\text {PLC}= 0.1$ and $C_t = 140$ $\mu$M. This choice simulates a closed-cell environment with an ER $\text {Ca}^{\text {2}+}$ concentration $c_e = 770$ $\mu$M. The attractor has an amplitude of $c \approx 0.2$ $\mu$M and period $T \approx 4$ seconds (Fig. 3B).
3. Broad spike: Parameter values $\delta = 0, V_\text {PLC}= 0.1$ $\mu$M/s and $C_t = 75$ $\mu$M. This choice simulates a closed-cell environment with an ER $\text {Ca}^{\text {2}+}$ concentration $c_e = 412$ $\mu$M. The attractor has an amplitude of $c \approx 0.4$ $\mu$M and period $T \approx 10$ seconds (Fig. 3C).
4. Broad spike with an oscillatory plateau: Parameter values $\delta = 0, V_\text {PLC}= 0.195$ $\mu$M/s and $C_t = 95$ $\mu$M. This choice simulates a closed-cell environment with an ER $\text {Ca}^{\text {2}+}$ concentration $c_e = 522$ $\mu$M. The attractor has an amplitude of $c \approx 0.4$ $\mu$M and period $T \approx 20$ seconds (Fig. 3D).

### Bifurcation Analysis of the Open-cell Model

**Fig. 4:**
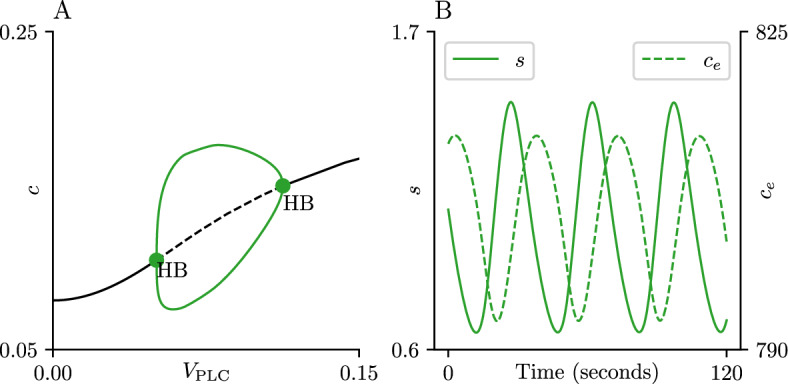
Open-cell bifurcation diagram. Panel A shows the bifurcation diagram for the open-cell model (1) using $V_\text {PLC}$ as a bifurcation parameter; the black curve represents the equilibrium solution, while the green solid lines represent the maximum and minimum *c* values of a branch of stable periodic orbits. The HB labels mark Hopf bifurcations of the equilibrium point. Panel B shows the *s* and $c_e$ traces in solid and dashed green lines, respectively, obtained from simulation of the open-cell model (1) with $V_\text {PLC}= 0.1$ and $\delta = 2$, as well as the parameters from Table 1 (Color figure online)

Figure 4 shows the bifurcation diagram and representative oscillatory traces obtained from simulation of the open-cell model (1), using $V_\text {PLC}$ as a bifurcation parameter, for a fixed value $\delta = 2$. The CRAC-mediated oscillations appear on the diagram as a branch of stable periodic orbits (green) situated between two Hopf bifurcations (HB). The distance between the Hopf bifurcations can be increased or decreased by modifying the $K_p$ parameter accordingly; this change does not affect the *c* values during oscillation, but does increase or decrease the mean $\text {IP}_{\text {3}}$ concentration, respectively.

For $V_\text {PLC}= 0.1$, the oscillation has a period of approximately 40 seconds and has a sinusoidal shape, with a period of slow increase in *c* followed by a period of slow decrease, both lasting approximately 20 seconds each. The maximal loss of ER $\text {Ca}^{\text {2}+}$ during a single oscillation is approximately 20 $\mu$M. This amount of ER depletion causes a constant, but moderate, formation of the CRAC channel, which causes *s* to oscillate between 25% to 50% of its maximal rate. The value of *c* oscillates from 0.1 to 0.15 approximately; the amplitude of this oscillation can be increased by rescaling *c*, but due to its involvement in most of the fluxes this would necessitate a substantial rescaling of the values in Table 1.

**Table 1:** Parameter values for model (1)

Parameter	Value	Parameter	Value
$V_\text {PM}$	3 $\mu$M/s	$\gamma$	5.5
$K_\text {PM}$	0.2 $\mu$M	$k_f$	1.6 $\mu$M
$V_\text {SOCE}$	3 $\mu$M	$k_\beta$	0.4
$s_1$	0.2	$K_p$	10 $\mu$M
$\tau _s$	15 s	$K_c$	0.16 $\mu$M
$K_e$	800 $\mu$M	$K_h$	0.168 $\mu$M
$V_\text {SERCA}$	2 $\mu$M/s	$\tau _\text {max}$	7.5 s
${\bar{K}}$	$1 \times 10^{-8}$	$K_\tau$	0.095
$K_\text {SERCA}$	0.19 $\mu$M	$\tau _p$	2 s
$V_\text {deg}$	6 $\mu$M/s	$K_\text {deg}$	0.5 $\mu$M

#### CRAC-mediated Oscillations Require a Steep and Delayed SOCE Mechanism

**Fig. 5:**
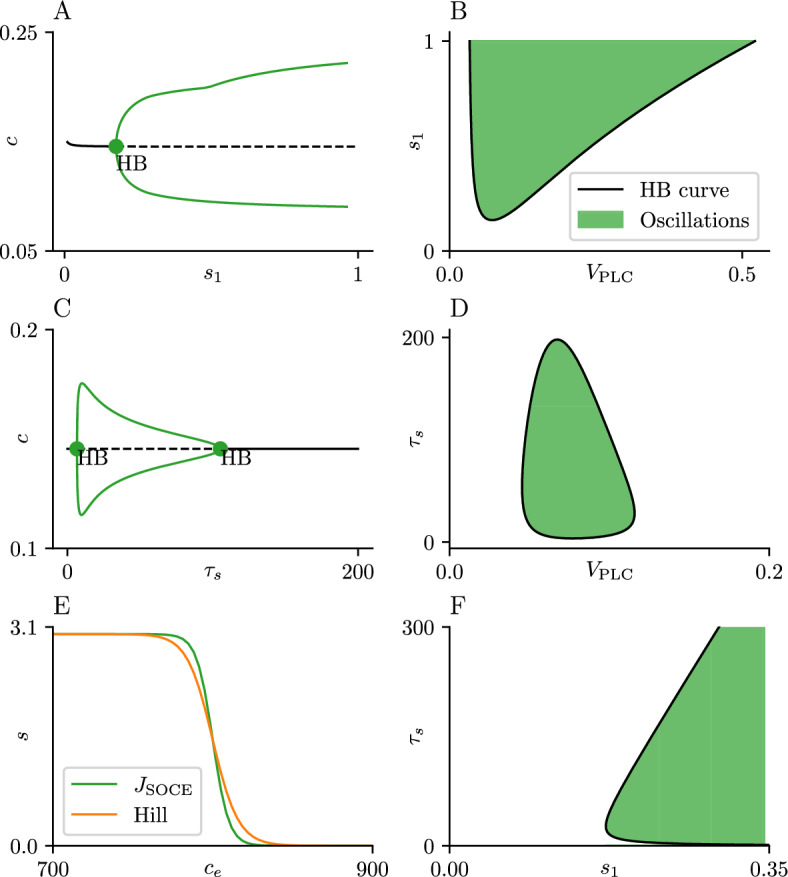
Open-cell bifurcation diagrams for steepness and delay parameters. Panel A shows the $s_1$-bifurcation diagram for the open-cell model (1) for $V_\text {PLC}= 0.1$ and $\delta = 2$. Panel C shows a similar diagram using $\tau _s$ as a bifurcation parameter. In both panels, the black curve represents the equilibrium solution, while the green solid lines show the maximum and minimum *c* values of a branch of stable periodic orbits. Panels B, D and F show the locations in the $s_1-V_\text {PLC}$, $\tau _s-V_\text {PLC}$ and $s_1-\tau _s$ (respectively) parameter planes of the Hopf bifurcations (black curves) and regions of CRAC-mediated oscillations (green-shaded regions). Panel E shows the SOCE activation curve with a steepness value $s_1 = 0.2$ in green, and a reverse Hill curve with a corresponding coefficient $n = 100$. Parameter values not specified here can be found in table 1 (Color figure online)

The oscillation shown in Fig. 4B ($V_\text {PLC}= 0.1$) uses a value of $s_1 = 0.2$. This choice of $s_1$ was made to ensure that $J_\text {SOCE}$ was steep enough as a function of $c_e$ for the CRAC-mediated oscillations to exist. This value can be decreased to ensure a shallower activation. To investigate the required steepness of this curve, we performed parameter continuation over $s_1$ on both the steady state and the stable periodic orbit observed with $V_\text {PLC}= 0.1$ (Fig. 5A). The branch of CRAC-mediated oscillations exists for $s_1 \ge 0.18$. Similar diagrams can be obtained for close values of $V_\text {PLC}$, but the exact position of the HB seen in Panel A will change. We performed two parameter continuation on this HB point and plot the corresponding curve in $s_1-V_\text {PLC}$ parameter space (Fig. 5B).

This HB curve divides the plane into two regions, one where CRAC-mediated oscillations are observed (shaded green) and one where no oscillations are observed. Continuation reveals a minimum value of $s_1$ needed to sustain the CRAC-mediated oscillations, given by $s_1 \approx 0.15$. This minimum value suggests that the formation of the CRAC channel in these cells follows a very steep ‘trigger-like’ activation curve. For comparison, our SOCE activation curve has a similar steepness as a reverse Hill function with a coefficient $n = 100$ (Fig. 5E).

A similar analysis is performed on the parameter $\tau _s$ (Fig. 5C, D). The time series shown in Fig. 3A uses a value of $\tau _s = 15$. CRAC-mediated oscillations are observed for a wide range of parameter values but, crucially, the diagram in Panel C shows the branch of stable periodic orbits ending at a HB at both high and low $\tau _s$ values. This observation suggests that, although a delayed SOCE activation is necessary to exhibit CRAC-mediated oscillations, this delay must be bounded for the oscillations exist. Panel D shows the curve obtained by following the two HBs in the $\tau _s-V_\text {PLC}$ parameter space. This curve once again divides the parameter plane into a region with CRAC-mediated oscillations and one with no response. This region is bounded, in contrast to the region obtained in the $s_1-V_\text {PLC}$ scenario, which further emphasizes the upper and lower bounds on the delay needed to observe the CRAC-mediated oscillations.

These two observations are not independent of each other; it could be argued that the same result could be generated by using a less steep curve alongside a faster formation timescale. To investigate this, we followed the HB point in the $s_1-\tau _s$ parameter space for a fixed value of $V_\text {PLC}= 0.1$ (Fig. 5F). While CRAC-mediated oscillations are present for a region with a less steep activation curve and a faster formation of the CRAC channel, the HB curve defines a positive boundary on both parameters, and no oscillations are observed for low values of $s_1$ and $\tau _s$, indicating that both features of our model are necessary for the existence of the CRAC-mediated oscillations.

#### $\text {IP}_{\text {3}}\text {R}$ based $\text {Ca}^{\text {2}+}$ Spikes are Suppressed by CRAC Activity

For the CRAC channel to form and allow the influx of extracellular $\text {Ca}^{\text {2}+}$ the ER must itself be depleted enough to activate the STIM proteins and create the STIM-Orai complex. In our model, this $\text {Ca}^{\text {2}+}$ release from the ER must necessarily be done through the $\text {IP}_{\text {3}}\text {R}$. The literature on the activity of the $\text {IP}_{\text {3}}\text {R}$ and its regulation by both $\text {Ca}^{\text {2}+}$ and $\text {IP}_{\text {3}}$ is extensive and a thorough review on the subject can be found in Foskett et al. (2007).

In our model, the release of $\text {Ca}^{\text {2}+}$ from the ER is controlled by the open probability of the channel ($P_0$). Once open, the $\text {Ca}^{\text {2}+}$ released from the channel activates the $\text {IP}_{\text {3}}\text {R}$ in a process known as $\text {Ca}^{\text {2}+}$-induced $\text {Ca}^{\text {2}+}$ release (CICR), which is the process responsible for the fast upswing in concentration observed in $\text {Ca}^{\text {2}+}$ spikes (Fig. 3B–D) (Dupont et al. 2016; Sneyd et al. 2017; Jelbart et al. 2022). After opening, the receptors are inactivated and remain closed for a time. The variable *h* represents the rate of activation of the $\text {IP}_{\text {3}}\text {R}$ in response to the binding of $\text {Ca}^{\text {2}+}$; the rate at which this activation occurs is regulated by *c* via modulation of the timescale $\tau _h$ (1c). Additionally, it is worth remarking that increases in *c* also promote degradation of $\text {IP}_{\text {3}}$ (1d).

**Fig. 6:**
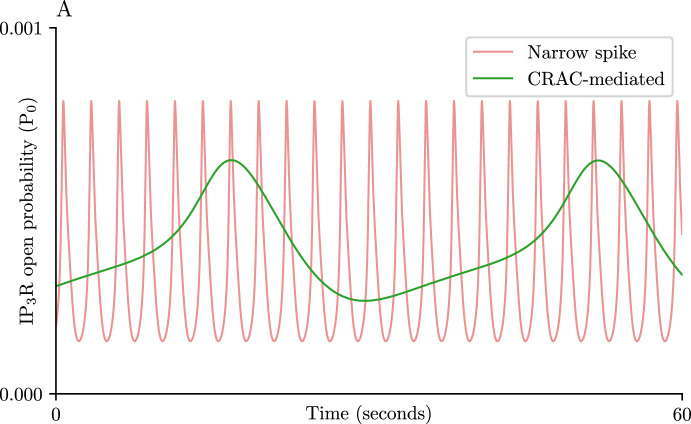
Modulation of $\text {IP}_{\text {3}}\text {R}$ activity by CRAC. Time series of the open probability of the $\text {IP}_{\text {3}}\text {R}$s during a CRAC-mediated oscillation ($\delta =2$) in green, and a similar series during a narrow spike ($\delta = 0$) in red. In both cases the time series were obtained by stimulating the open-cell model (1) with $V_\text {PLC}= 0.1$. Parameter values not specified here can be found in table 1 (Color figure online)

**Fig. 7:**
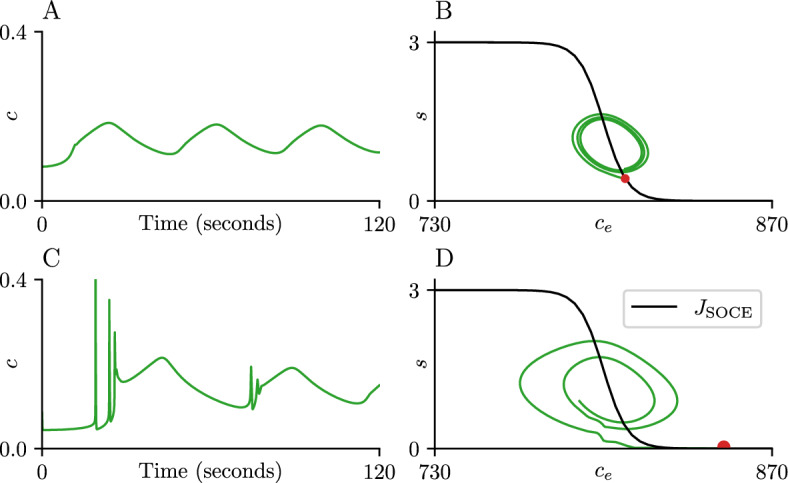
Initial high loads of ER $\text {Ca}^{\text {2}+}$ lead to transient narrow spikes. Panels show stimulation of the open-cell model (1) (represented by an increase in $V_\text {PLC}$ from 0 to 0.1) under different initial $c_e$ values. The corresponding initial *c* and *s* values are obtained by solving equations 1b and 1e assuming the cell is unstimulated ($V_\text {PLC}= 0$). The top panels use initial values $c = 0.809$, $c_e = 809$, $s = 0.421$, while the bottom panels use $c = 0.850$, $c_e = 850$, $s = 0$. Panels A and C show the time series for the cytoplasmic $\text {Ca}^{\text {2}+}$ concentration (*c*), panels B and D the $J_\text {SOCE}$ curve (4) as a function of $c_e$ alongside a projection of the corresponding trajectory on the $s-c_e$ plane. The initial conditions are marked with a red dot for visibility. All panels use a value of $\delta = 2$. Parameter values not specified here can be found in table 1 (Color figure online)

Inspection of the time series for the $P_0$ term during the CRAC-mediated oscillation reveals that no fast CICR is observed during this scenario, in contrast with the similar time series for a narrow spike (Fig. 6). The mathematical expression for the $P_0$ term can be found in Appendix A. Instead of a fast opening and closing of the channel, the $P_0$ curve indicates that the $\text {IP}_{\text {3}}\text {R}$ channel remains open for a longer time which allows for a gradual $\text {Ca}^{\text {2}+}$ leak from the ER on the timescale of the CRAC dynamics. This gradual leak allows for greater depletion of the ER stores during CRAC-mediated oscillations when compared to the narrow spikes (Fig. 10B-D).

The effect of the suppression of the fast CICR by CRAC activity can be captured by simulating the open-cell model (1) under two different sets of initial conditions, a ‘low’ ER load ($c_e = 809$) such that CRAC is partially activated at the start of the simulation (Fig. 7A-B), and a ‘high’ ER load ($c_e = 850$). The initial conditions for *c* and *s* are obtained by solving equations 1b and 1e assuming an unstimulated cell ($V_\text {PLC}= 0$). Under these conditions, CRAC is completely inactivated at the start of the simulation in the high ER load scenario (Fig. 7C-D). The time series of the cytoplasmic $\text {Ca}^{\text {2}+}$ in the low ER load does not exhibit transient spiking as it converges to the limit cycle. The relatively large value of *s* positions this initial state close to the limit cycle (Fig. 7B). The figure also shows the $J_\text {SOCE}$ curve as a function of $c_e$. As the $c_e$ load decreases and crosses the midpoint of the $J_\text {SOCE}$ curve ($K_e = 800$ $\mu$M) it triggers the activation of CRAC which increases the value of *s*. Superimposing this trajectory on the $J_\text {SOCE}$ activation curve shows $c_e$ oscillating between 790 and 820 $\mu$M, which correspond to the complete activation ($J_\text {SOCE}= 3$) and complete deactivation ($J_\text {SOCE}= 0$) values of the curve, respectively. However, due to the slow evolution of *s*, the actual flux though the channel never reaches either of these values.

In the high ER load scenario, the time series for *c* shows transient $\text {Ca}^{\text {2}+}$ spikes before converging to the limit cycle. These spikes are less notable when looking at the $c_e-s$ plane, due to the relatively low amount of ER $\text {Ca}^{\text {2}+}$ being released during this type of oscillation. The spikes persist for approximately 30 s, a time frame similar to the formation of the CRAC channel, before being replaced by the CRAC-mediated oscillations once the channel has been formed. These narrow spikes are only observed at values of $c_e$ close to the midpoint of the $J_\text {SOCE}$ curve ($K_e$). Using the expression for total $\text {Ca}^{\text {2}+}$, $C_t = c + c_e/\gamma$, we can notice that these transient spikes appear at $C_t$ values between 140 and 150 $\mu$M. Narrow spikes are the only type of oscillation observed in the closed-cell model for this range of $C_t$ values (Sect. 3.3). Thus, the model suggests that restricting extracellular exchange during a CRAC-mediated oscillation could result in the appearance of narrow spikes, but not wide spikes.

### Bifurcation Analysis of the Closed-Cell Model

**Fig. 8:**
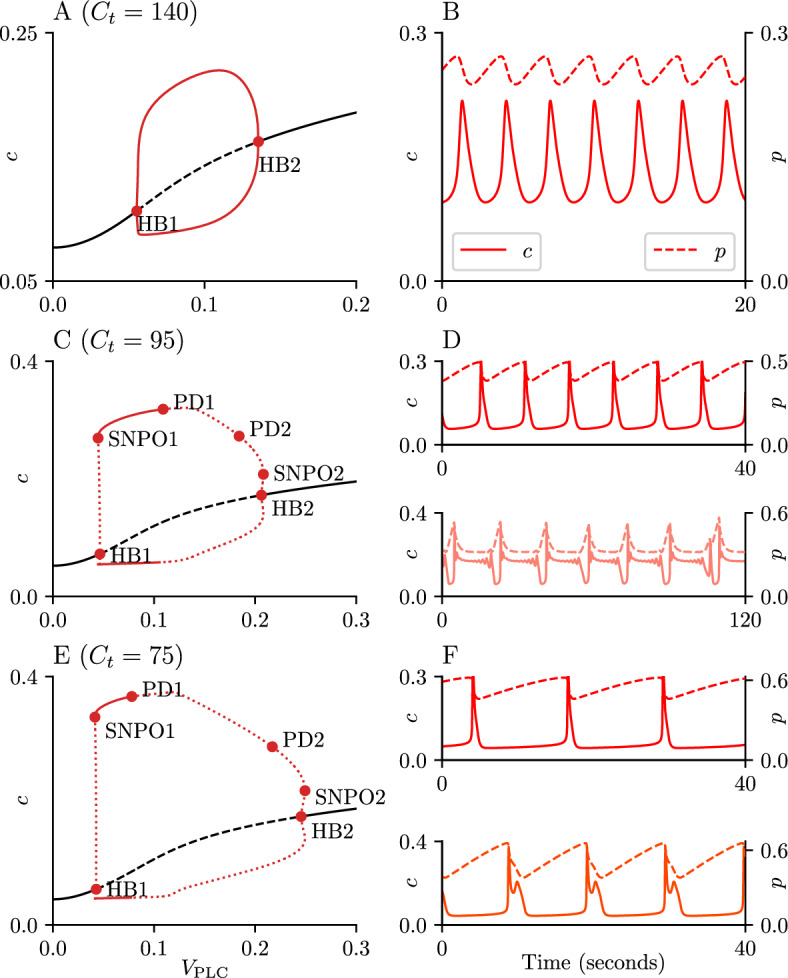
Closed-cell model bifurcation diagram. Panel A shows the complete bifurcation diagram of the closed-cell model (2) using $V_\text {PLC}$ as a bifurcation parameter, for $C_t = 140$. Panels C and E show similar (partial) bifurcation diagrams for $C_t = 95$ and $C_t = 75$, respectively. The black curve represents the equilibrium solution, while the red lines show the maximum and minimum values of *c* on the branches of periodic orbits; stable orbits are represented by solid lines and unstable orbits by dashed lines. Panels B, D and F show the time series for the cytoplasmic $\text {Ca}^{\text {2}+}$ concentration in solid lines, and the cytoplasmic $\text {IP}_{\text {3}}$ concentration in dashed lines. Panel B shows the attractor obtained for parameter values $V_\text {PLC}= 0.1, C_t = 140$. Panel D shows two types of attractors obtained for $C_t = 95$, for $V_\text {PLC}= 0.09$ (top) and $V_\text {PLC}= 0.195$ (bottom). Panel F shows two types of attractors obtained for $C_t = 75$, for $V_\text {PLC}= 0.06$ (top) and $V_\text {PLC}= 0.1$ (bottom). Parameter values not specified here can be found in table 1 (Color figure online)

To study the narrow-spike oscillations generated by the closed-cell model (2), we compute bifurcation diagrams with $V_\text {PLC}$ as the bifurcation parameter and various choices of fixed $C_t$. The case for $C_t = 140$ is shown in Fig. 8A. Here, there are two supercritical Hopf bifurcations with a branch of stable periodic solutions between these bifurcations. Corresponding time series for *c* and *p* for a representative value of $V_\text {PLC}$ are shown in Fig. 8B.

When $C_t = 95$ the two HBs are subcritical, and four new bifurcation points are detected: two saddle-nodes of periodic orbits (SNPO) and two periodic doubling (PD) bifurcations. The branch of periodic solutions is now only stable for parameter values between the SNPO1 and the PD1 bifurcations (Fig. 8C) A representative time series corresponding to the stable branch of periodic solutions is shown in the upper part of Panel D. Secondary branches of periodic solutions are created from the PDs, but they are not plotted here. The region between the PD2 and the HB2 points has no attractors on either the equilibrium line or the branch of periodic orbits. Instead, the corresponding attractor occurs on a branch of periodic solutions that is isolated from the rest of the diagram and is not plotted here. A representative time series for this region can be seen in the lower part of Panel D, which corresponds to the wide spike with an oscillatory plateau shown in Fig. 3.

**Fig. 9:**
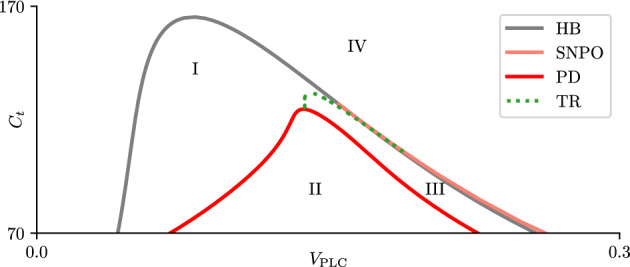
Two-parameter bifurcation set for the closed-cell model. The black line represents a curve of Hopf bifurcations (HB); the red line a curve of period doubling bifurcations (PD); the orange line represents a curve of saddle nodes of periodic orbits (SNPO) emanating from the right branch of the HB curve; the dotted green line represents a curve of Neimark-Sacker bifurcations (TR). Labels I, II, III and IV indicate the approximate region in parameter space where narrow spikes, wide spikes, wide spikes with plateau, and no oscillations are observed, respectively. Parameter values not specified here can be found in table 1 (Color figure online)

The diagram obtained at $C_t = 75$ shares all the qualitative features of the $C_t = 95$ case. However, the stable region of the main periodic orbit branch has shrunk. The top part of Panel F shows a representative trace from the stable region of the main branch of periodic orbits, between the SNPO1 and PD1 points of Panel E@; the lower part shows an attractor belonging to a different branch of stable periodic orbits that emanates from the main branch at the PD2 point.

The information about the different types of oscillations produced by the closed-cell model (2) can be summarized by looking at the two parameter bifurcation set shown in Fig. 9. Narrow spike oscillations can be found in the region bounded by the HB, PD and TR curves (Region I). In reality the left boundary is set by a curve of SNPO that lies very close to the HB curve, but it is not drawn here for simplicity.

Delimiting the regions in parameter space where the wide spikes and wide spikes with plateau exist is a harder task. The PD curve in Fig. 9 can be used as proxy for the boundary of the region containing wide spikes (Region II) due to it separating the region where the narrow spikes are stable, but it must be remarked that this curve is not the actual boundary. We have found wide spikes with oscillatory plateaus in the area bounded by the PD, TR and SNPO curves (Region III). It is unlikely that these curves are the exact boundaries between Regions I, II and III, but they are close to the boundaries and provide useful proxies for distinguishing the regions in which the two types of wide spikes are observed.

### Qualitative Differences Between the Two Families of Oscillations

**Fig. 10:**
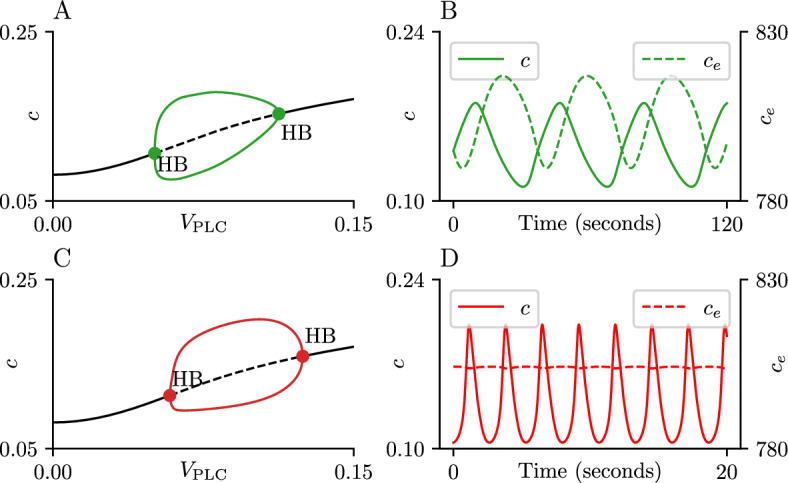
$\text {IP}_{\text {3}}\text {R}$ and CRAC-mediated oscillations on the open-cell model. Panel A shows the bifurcation diagram for the open-cell model (1) using $V_\text {PLC}$ for a fixed value of $\delta = 2$. Panel B shows the time series obtained by stimulating the model with $V_\text {PLC}= 0.1$, the cytoplasmic $\text {Ca}^{\text {2}+}$ concentration is plotted as a solid curve, and the ER concentration as a dashed curve. Panels C and D show analogous figures, but with a fixed parameter value of $\delta = 0.01$. Parameter values not specified here can be found in Table 1

For $\delta \approx 2$, the open-cell model (1) exhibits CRAC-mediated oscillations. In contrast, for $\delta \approx 0$ the model exhibits $\text {IP}_{\text {3}}\text {R}$ mediated $\text {Ca}^{\text {2}+}$ spikes. These two behaviours are shown in Panels B and D of Fig. 10, where we compare the attractor obtained for $\delta = 2$ against the corresponding attractor for $\delta = 0.01$. The CRAC-mediated oscillation has a longer period than the $\text {IP}_{\text {3}}\text {R}$ mediated spike, as well as a wide variation in ER concentration values, needed to trigger the formation of the CRAC channel.

Bifurcation diagrams for examples of these two cases are shown in Panels A and C of the same figure. While the overall structure of the two bifurcation diagrams is the same, the time series show there are significant differences in the behaviour of the attracting periodic solutions.

**Fig. 11:**
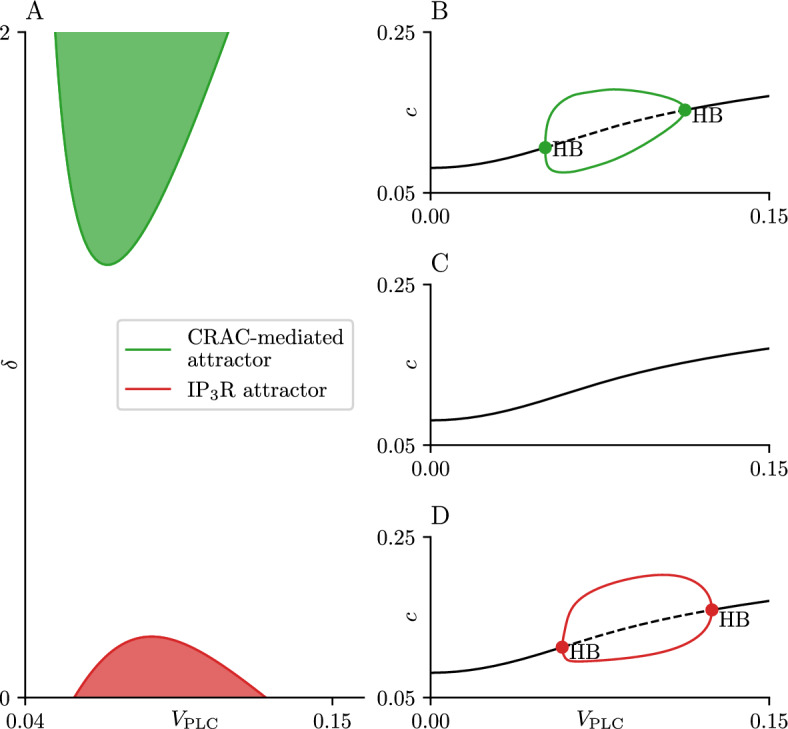
Two-parameter bifurcation set for the open-cell model. Panel A shows the two-parameter bifurcation set for varying $\delta$ and $V_\text {PLC}$. The border of the upper green region represents a curve of HB observed for high values of $\delta$, while the green region itself represents the parameter region where the CRAC-mediated attractor is observed. The border of the red region is a curve of HB observed at $\delta$ values close to zero. Panels B, C and D show the bifurcation diagrams of the open-cell model (1) using $V_\text {PLC}$ as a bifurcation parameter, for fixed values $\delta = 2, 0.62, 0.01$, respectively. The black curve represents the equilibrium solution, while the red and green lines represent branches of periodic orbits. Stable attractors are represented by solid lines and unstable attractors by dashed lines. Parameter values not specified here can be found in table 1 (Color figure online)

**Fig. 12:**
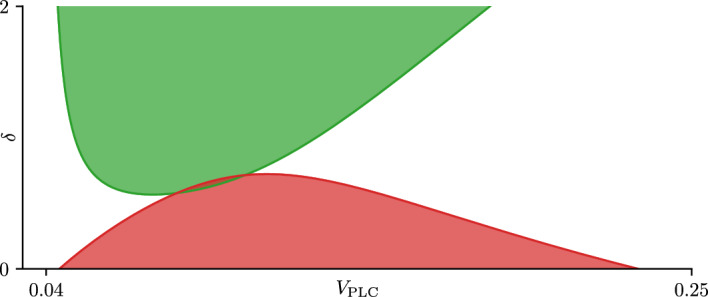
Two-parameter bifurcation set for the open-cell model with a smaller CRAC activation threshold. Here $K_e = 400$. This figure uses the colouring scheme of Fig. 11. Parameter values not specified here can be found in table 1 (Color figure online)

To explain the transition between the CRAC-mediated and the $\text {IP}_{\text {3}}\text {R}$-mediated oscillations in the open-cell model (1) we treat $\delta$ as a bifurcation parameter and perform two parameter continuation on the HB points found on both the $\delta = 2$ and the $\delta = 0.01$ diagrams (Panels A and C of Fig. 10). The resulting diagram shows two non-intersecting curves of HB (Fig. 11A). These curves define two regions of space, one where the CRAC-mediated oscillations are observed (shaded green) and one where $\text {IP}_{\text {3}}\text {R}$-mediated oscillations are observed (shaded red). At parameter values outside these two regions, no stable periodic orbits are found in the model. Bifurcation diagrams for three representative fixed values of $\delta$ are shown in panels B-D.

These two regions can be made to intersect by decreasing the parameter $K_e$ from 800 $\mu$M to 400 $\mu$M (Fig. 12A). This change causes both the green and red regions to grow in parameter space and to intersect at $\delta \approx 0.6$. Physiologically, lowering $K_e$ reduces the activation threshold for the CRAC channel, with in turn causes the resting ER $\text {Ca}^{\text {2}+}$ load to decrease. At $\delta = 2$, the $V_\text {PLC}$ bifurcation diagram exhibits the same features as the analogous diagram for $K_e = 800$ $\mu$M.

At the other end of the diagram ($\delta = 0.01$) the structure of the 1-parameter $V_\text {PLC}$ bifurcation diagram differs from that for the case $K_e = 800$ $\mu$M. It is worth stressing that at small values of $\delta$ the behaviour of the open-cell model can be approximated by that for the closed-cell model for an appropiate value of $C_t$. The $K_e = 800$ case shows a $V_\text {PLC}$ diagram (Fig. 10C) qualitatively similar to the one generated by the closed-cell model with a value of $C_t = 140$ (Fig. 8A). Meanwhile, the $V_\text {PLC}$ diagram for the $K_e = 400$ $\mu$M case is qualitatively closer to the closed-cell bifurcation diagram corresponding to $C_t = 75$ (Fig. 8E), with both of them containing branches of wide and narrow spikes. A detailed investigation of any interaction between the red and green regions as $\delta$, among other system parameters, varies is left for future work.

## Discussion

Literature on $\text {Ca}^{\text {2}+}$ dynamics inside lymphocytes is extensive, and experimental insight about the particular type of $\text {Ca}^{\text {2}+}$ oscillations observed in Fig. 1 dates back forty years and has highlighted the importance of SOCE through the CRAC channel for the oscillations to persist with time (Zweifach and Lewis 1995; Dolmetsch and Lewis 1994). Benson et al. have provided experimental evidence to corroborate this hypothesis, as well as evidence for a second type of oscillation that emerges in the absence of SOCE@. With this in mind, we have created a mathematical model based on physiological principles that can reproduce both the CRAC-mediated and $\text {IP}_{\text {3}}\text {R}$-mediated $\text {Ca}^{\text {2}+}$ oscillations.

In cells without extracellular $\text {Ca}^{\text {2}+}$ exchange, the closed-cell model (2) is able to generate narrow spikes, simple wide spikes and wide spikes with an oscillatory plateau, depending on the rate of $\text {IP}_{\text {3}}$ production due to $\text {PLC}$ activity and a degradation rate dependent on the cytoplasmic $\text {Ca}^{\text {2}+}$ concentration. Positive feedback mechanisms relying on phospholipase C (PLC) activity on *c* were also explored but were unable to generate oscillations with oscillatory plateaus (Lee et al. 2023).

Regarding the broad spikes with oscillatory plateaus, these solutions were observed near the right HB in the case $C_t = 95$, and below belong to an isolated branch of periodic solutions located near the HB2 point on the diagram (Fig. 8D). While it is possible to numerically observe this branch by performing continuation on the periodic solution, we do not have a working hypothesis for the onset of this branch or the connection it might have to the main branch emanating from the HB@. We believe that the onset of this branch would require the use of slow-fast analysis on the model, a technique that is outside the scope of this paper.

The role of the parameter $C_t$ in the closed-cell model is also worth emphasizing. The model predicts that lower values of total $\text {Ca}^{\text {2}+}$ in the cell give rise to wide spike oscillations and reduces the parameter range where narrow spike oscillations are observed. This prediction could help to explain the changes in the behaviour of the response seen in the $\text {Ca}^{\text {2}+}$-free experiments (Fig. 1); with the absence of a refilling mechanism, cells will empty their $\text {Ca}^{\text {2}+}$ stores and this is accompanied by a transition in the $\text {Ca}^{\text {2}+}$ response, from a narrow spike to a wide spike with (or without) an oscillatory plateau.

In cells with extracellular $\text {Ca}^{\text {2}+}$ exchange, the open-cell model (1) is capable of reproducing the CRAC-mediated oscillations observed experimentally. The time series of the open probability of the $\text {IP}_{\text {3}}\text {R}$ (Fig. 6) during such an oscillation highlights the gradual $\text {IP}_{\text {3}}\text {R}$ activity happening in the background of the strong SOCE signal, where fast-CICR has been suppressed. This finding is supported by prior experiments that were able to induce CRAC-mediated oscillations in cells by the blockage of SERCA pumps with Tg, causing a constant, but small, $\text {Ca}^{\text {2}+}$ leak from the ER (Dolmetsch and Lewis 1994). The time series presented in Fig. 6 suggests that, under TCR stimulation, the $\text {IP}_{\text {3}}\text {R}$ of these cells act in a similar fashion, causing a slow but constant leak of $\text {Ca}^{\text {2}+}$ just strong enough to induce the formation of the CRAC channel and induce SOCE@.

Our model introduces a new variable *s* which represents the $\text {Ca}^{\text {2}+}$ influx through the CRAC channel. The activation curve for the CRAC channel in our model needs to be rather steep for these oscillations to occur. The formation of this channel also needs to be done over a sufficiently long period to allow the ER to refill and deplete enough so that the oscillations can regenerate. This time delay on the formation of the channel can be attributed to the time needed to activate the STIM isoforms and for these to move away from the ER membrane and closer to the Orai isoforms on the PM (Trebak and Kinet 2019). We have shown that the steepness and the time delay are both necessary in our model to exhibit the CRAC-mediated oscillations. Our model currently does not make a distinction between STIM1-mediated and STIM2-mediated SOCE@. Experimental evidence suggests that the two STIM molecules have a similar level of importance in this cell type, with STIM1 being slightly more important (Benson et al. 2023). Benson et al. have also performed experiments in cells without STIM1 or STIM2, and found that no CRAC-mediated response was obtained in cases where at least one STIM protein was missing. Our $J_\text {SOCE}$ activation curve could be modified to account for different types of STIM and investigate the lack of a CRAC-mediated oscillation in those cells.

The CRAC-mediated oscillations generated by the open-cell model (1) also replicate the behaviour initially hypothesized by Dolmetsch and Lewis (Dolmetsch and Lewis 1994), who predicted that the ER $\text {Ca}^{\text {2}+}$ concentration and $\text {Ca}^{\text {2}+}$ influx oscillate out of phase with each other, as seen in Fig. 4. Exploration of the corresponding time series of the $\text {IP}_{\text {3}}\text {R}$ and $\text {SERCA}$ fluxes suggest that $\text {Ca}^{\text {2}+}$ transport through the ER membrane also oscillates with the same period, overriding the fast CICR from the $\text {IP}_{\text {3}}\text {R}$. We conjecture then that the appearance of CRAC-mediated oscillations is dependent on the timescales of SOCE activation ($\tau _s$) and activation of the $\text {IP}_{\text {3}}\text {R}$ ($\tau _\text {max}$) being on the same order of magnitude. Experimentally, this can be seen in the fact that increasing either $\tau _s$ from 15 to 150 or $\tau _\text {max}$ from 7.5 to 75 both show no oscillations for any $V_\text {PLC}$ value, but increasing both at the same time does produce a CRAC-mediated oscillation (not shown here). This conjecture is not explored further in this paper and is left for future work.

The presence of the CRAC-mediated oscillation is also dependent on $\delta$, as seen in Figs. 11 and 12, which can be interpreted as a dependence on the PM fluxes being ‘fast enough’ in comparison to the ER fluxes. A proper discussion on the relative speed of the PM fluxes, however, necessitates a proper non-dimensionalization of the model, which is left for future work.

Finally, we have shown that the oscillations observed in the presence and absence of extracellular $\text {Ca}^{\text {2}+}$ have fundamentally different dynamical properties. One of the primary distinctions between the two families of oscillations is the amount of $\text {Ca}^{\text {2}+}$ transported through the ER during oscillation; in narrow spikes the ER stores are only slightly depleted, with a total loss in $\text {Ca}^{\text {2}+}$ concentration no greater that 5 $\mu$M@; in CRAC-mediated oscillations, the ER stores lose up to 30 $\mu$M of $\text {Ca}^{\text {2}+}$. This difference in the magnitude of $\text {Ca}^{\text {2}+}$ loss from the ER is not visible from the cytoplasmic $\text {Ca}^{\text {2}+}$ concentration, which has the same order of magnitude in both narrow spikes and CRAC-mediated oscillations.

We showed that the $\text {IP}_{\text {3}}\text {R}$-based narrow spikes and the CRAC-mediated oscillations belong to different families of stable periodic orbits that can both be found in the open-cell (1) under the correct parameter conditions. We have found it possible to intersect the two curves of HB seen in Fig. 11 by varying the parameter $K_e$. In this intersecting region, two branches of periodic orbits can coexist, which further emphasized their differing nature. We have not been able to ‘pull down’ the HB curve associated to the CRAC-mediated oscillations towards the boundary $\delta = 0$, however, which emphasizes the dependence that this oscillation type has on the relative speed of the PM fluxes. It is worth remarking that these two families of oscillations can be found in the open-cell model simply by varying the parameter $\delta$, with CRAC-mediated oscillations present at ‘high’ values of the parameter, and narrow spike oscillations present at ‘low’ values (Fig. 11). This switch in the type of oscillation implies that changes in $\delta$ are able to modulate the rate of $\text {Ca}^{\text {2}+}$ exchange through the ER membrane during an oscillation. The rate of transport through the PM is known to affect the frequency of oscillations in $\text {Ca}^{\text {2}+}$ models (Dupont et al. 2016; Sneyd et al. 2004).

There is no intrinsic reason why the two oscillation types could not ‘coexist’ on the same periodic solution. In fact, the open-cell model can exhibit a hybrid oscillation where a narrow spike appears on top of a CRAC-mediated oscillation using the parameter values $K_e = 400$ $\mu$M, $\delta = 0.62$ and $V_\text {PLC}= 0.1$ $\mu$M alongside the values shown in Table tab:BurstOpenCell. We did not include this type of orbit in the paper as it did not correspond to any of the experimental traces observed. The hypothesis that this narrow spike occurs as one of our variables crosses an activation threshold (the main candidates being *c* and *h*) is something have explored, but confirming that hypothesis would require the use of mathematical techniques that exceed the scope of this work.

## Supplementary information

The code used to generate the figures can be found in GitHub (0Huitzil 2023).

## Appendix A Flux expressions & parameter values

The open-cell version of the model has corresponding equations:

A1a $$\begin{aligned} \frac{dc}{dt}&= J_{\text {IP}_{\text {3}}\text {R}}(c, c_e, h, p) - J_{\text {SERCA}}(c, c_e) + \delta (s - J_{\text {PM}}(c)), \end{aligned}$$

A1b $$\begin{aligned} \frac{dc_e}{dt}&= \gamma (J_{\text {SERCA}}(c, c_e) - J_{\text {IP}_{\text {3}}\text {R}}(c, c_e, h, p)) , \end{aligned}$$

A1c $$\begin{aligned} \tau _h(c) \frac{dh}{dt}&= h_\infty (c) - h,\end{aligned}$$

A1d $$\begin{aligned} \tau _p \frac{dp}{dt}&= V_\text {PLC}- J_\text {deg}(c),\end{aligned}$$

A1e $$\begin{aligned} \tau _s \frac{ds}{dt}&= J_\text {SOCE}(c_e) - s. \end{aligned}$$

The flux expressions are given by

A2a $$\begin{aligned} J_\text {SERCA}&= V_s \frac{c^2 - {\bar{K}}c_e^2}{c^2 + K_s^2}, \end{aligned}$$

A2b $$\begin{aligned} J_\text {PM}&= V_\text {PM}\frac{c^2}{c^2 + K_\text {PM}^2},\end{aligned}$$

A2c $$\begin{aligned} J_\text {SOCE}&= \frac{V_\text {SOCE}}{1 + e^{s_1(c_e - K_e)}}, \end{aligned}$$

A2d $$\begin{aligned} J_\text {deg}&= V_\text {deg}\frac{c^2}{c^2 + K_\text {deg}^2} p, \end{aligned}$$

A2e $$\begin{aligned} h_\infty&= \frac{K_h^4}{K_h^4 + c^4}, \end{aligned}$$

A2f $$\begin{aligned} \tau _h&= \tau _\text {max}\frac{K_\tau ^4}{K_\tau ^4 + c^4}, \end{aligned}$$

A2g $$\begin{aligned} J_{\text {IP}_{\text {3}}\text {R}}&= k_f P_0(c,p,h)(c_e - c), \end{aligned}$$

where $P_0$ is the open probability of the $\text {IP}_{\text {3}}\text {R}$ and is based on a modal model of the receptor described in Dupont et al. (2011). $P_0$ has the form

A3 $$\begin{aligned} P_0&= \frac{\beta }{\beta + k_\beta (\beta + \alpha )}. \end{aligned}$$

with $\alpha$ and $\beta$ given by

A4 $$\begin{aligned} \alpha&= A(p) (1 - m_\alpha (c) h_\alpha (c)), \end{aligned}$$

A5 $$\begin{aligned} \beta&= B(p) m_\beta (c) h. \end{aligned}$$

The model is further simplified by assuming $m_\alpha = m_\beta$ and $A(p) = 1 - B(p)$, which reduces the number of parameters while retaining the overall structure of the $\alpha$ and $\beta$ functions. Finally, we define

A6 $$\begin{aligned} m_\alpha&= \frac{c^4}{K_c^4 + c^4},\end{aligned}$$

A7 $$\begin{aligned} A(p)&= \frac{p^2}{K_p^2 + c^2} \end{aligned}$$

The closed-cell version of the model has corresponding equations

A8a $$\begin{aligned} \frac{dc}{dt}&= J_{\text {IP}_{\text {3}}\text {R}}(c, p, h) - J_{\text {SERCA}}(c) , \end{aligned}$$

A8b $$\begin{aligned} \tau _h(c)\frac{dh}{dt}&= h_\infty (c) - h, \end{aligned}$$

A8c $$\begin{aligned} \tau _p \frac{dp}{dt}&= V_\text {PLC}- J_\text {deg}(c). \end{aligned}$$

This version of the model makes use of the conserved quantity $C_t$ to write $c_e$ as a function of *c* given by

A9 $$\begin{aligned} c_e&= \gamma (C_t - c). \end{aligned}$$

The flux expressions are different to those used in the open-cell model because of this change, and are instead given by

A10a $$\begin{aligned} J_\text {SERCA}&= V_s \frac{c^2 - {\bar{K}}{\gamma (C_t - c)}^2}{c^2 + K_s^2}, \end{aligned}$$

A10b $$\begin{aligned} J_\text {deg}&= V_\text {deg}\frac{c^2}{c^2 + K_\text {deg}^2} p, \end{aligned}$$

A10c $$\begin{aligned} h_\infty&= \frac{K_h^4}{K_h^4 + c^4}, \end{aligned}$$

A10d $$\begin{aligned} \tau _h&= \tau _\text {max}\frac{K_\tau ^4}{K_\tau ^4 + c^4}, \end{aligned}$$

A10e $$\begin{aligned} J_{\text {IP}_{\text {3}}\text {R}}&= k_f P_0(c,p,h)(\gamma (C_t - c) - c), \end{aligned}$$

The parameter values used in the figures shown in this work can be found in the following table, except for $$V_\text {PLC}, \delta $$ and $$ C_t$$, in which case the relevant values can be found in the corresponding captions.
